# Addressing Reported Pro-Apoptotic Functions of NF-κB: Targeted Inhibition of Canonical NF-κB Enhances the Apoptotic Effects of Doxorubicin

**DOI:** 10.1371/journal.pone.0006992

**Published:** 2009-09-10

**Authors:** Brian K. Bednarski, Albert S. Baldwin, Hong Jin Kim

**Affiliations:** 1 Lineberger Comprehensive Cancer Center, University of North Carolina, Chapel Hill, North Carolina, United States of America; 2 Department of Surgery, University of North Carolina, Chapel Hill, North Carolina, United States of America; Roswell Park Cancer Institute, United States of America

## Abstract

The ability of the transcription factor NF-κB to upregulate anti-apoptotic proteins has been linked to the chemoresistance of solid tumors to standard chemotherapy. In contrast, recent studies have proposed that, in response to doxorubicin, NF-κB can be pro-apoptotic through repression of anti-apoptotic target genes. However, there is little evidence analyzing the outcome of NF-κB inhibition on the cytotoxicity of doxorubicin in studies describing pro-apoptotic NF-κB activity. In this study, we further characterize the activation of NF-κB in response to doxorubicin and evaluate its role in chemotherapy-induced cell death in sarcoma cells where NF-κB is reported to be pro-apoptotic. Doxorubicin treatment in U2OS cells induced canonical NF-κB activity as evidenced by increased nuclear accumulation of phosphorylated p65 at serine 536 and increased DNA–binding activity. Co-treatment with a small molecule IKKβ inhibitor, Compound A, abrogated this response. RT–PCR evaluation of anti-apoptotic gene expression revealed that doxorubicin-induced transcription of cIAP2 was inhibited by Compound A, while doxorubicin-induced repression of other anti-apoptotic genes was unaffected by Compound A or siRNA to p65. Furthermore, the combination of doxorubicin and canonical NF-κB inhibition with Compound A or siRNA to p65 resulted in decreased cell viability measured by trypan blue staining and MTS assay and increased apoptosis measured by cleaved poly (ADP-ribose) polymerase and cleaved caspase 3 when compared to doxorubicin alone. Our results demonstrate that doxorubicin-induced canonical NF-κB activity associated with phosphorylated p65 is anti-apoptotic in its function and that doxorubicin-induced repression of anti-apoptotic genes occurs independent of p65. Therefore, combination therapies incorporating NF-κB inhibitors together with standard chemotherapies remains a viable method to improve the clinical outcomes in patients with advanced stage malignancies.

## Introduction

Nuclear Factor-κB (NF-κB) plays a major role in a number of oncogenic processes, including development, metastasis and treatment outcomes [1]–[3]. This family of evolutionarily conserved transcription factors (p65 or RelA, p50/p105, p52/p100, RelB and cRel), which share a common Rel homology domain, typically exists as homo- or hetero-dimers in the cytoplasm where they are bound by inhibitory κB proteins (IκB), such as IκBα. In response to a variety of stimuli, the inhibitory κB kinase (IKK) complex, consisting of two catalytic subunits (IKKα and IKKβ) and a regulatory subunit (IKKγ/NEMO), can phosphorylate the IκB proteins targeting them for degradation by the 26S proteasome. As a result, NF-κB is released and can translocate to the nucleus to modulate gene transcription. A number of target genes have been identified, including anti-apoptotic proteins, proteins involved in angiogenesis, and proteins regulating cellular proliferation [2], [3].

The activation of anti-apoptotic gene transcription by NF-κB has been linked to the ability of malignancies to resist the cytotoxic effects of standard chemotherapeutics. Previous work from our laboratory and others has demonstrated that NF-κB is activated in response to a number of chemotherapies and irradiation [4]. Specifically, in fibrosarcoma cells, the induction of NF-κB activity by etoposide resulted in increased expression of A1/Bfl-1 while inhibition of NF-κB blocked the induction of A1/Bfl-1 and resulted in enhanced etoposide-induced cell death [5]. Moreover in colon cancer cells, NF-κB inhibition combined with CPT-11 (active metabolite of camptothecin) resulted in decreased xenograft growth when compared to chemotherapy alone [6], [7]. Similar effects of NF-κB inhibition have also been demonstrated in lung cancer and breast cancer [8], [9]. These studies collectively support an important role for NF-κB in the chemoresistance of solid tumors.

However, some recent reports have challenged this model and proposed that NF-κB activity seen in response to DNA damage induced by ultraviolet radiation and chemotherapeutics can function to promote cell death [10]–[12]. The most common stimuli used to reportedly induce pro-apoptotic NF-κB activity are the anthracycline, doxorubicin, and its analogues [10]–[12]. Two distinct mechanisms have been proposed to mediate this effect. Campbell et al. demonstrated that in osteosarcoma cells daunorubicin induces recruitment of NF-κB together with histone deacetylases to silence the transcription of Bcl-xL [11]. On the other hand, Ho et al. demonstrate that treatment of breast cancer cells with doxorubicin generates an NF-κB complex that is deficient in both phosphorylation and acetylation and represses anti-apoptotic gene transcription in a mechanism independent of histone deacetylases [12]. These studies further suggest that NF-κB activation may be required for doxorubicin to induce cell death and therefore that combining targeted NF-κB inhibition could actually serve to counteract the desired cell killing effects of chemotherapy [11], [12]. While these two studies describe mechanisms by which NF-κB can silence the expression of selective genes, there is little evidence in the literature demonstrating that inhibition of NF-κB activity renders chemotherapy less effective.

Answering this question is critical to determining the potential benefits or hazards to employing NF-κB inhibition as an adjunct to standard chemotherapy. Currently, the primary chemotherapy treatment of advanced stage sarcomas is doxorubicin, which only yields a 15–35% response rate [13], [14]. This dismal response, in combination with the fact that the therapeutic options have not changed in 20 years, highlights the highly chemoresistant nature of this disease [13]. As such, we employed the osteosarcoma model used by Campbell et al. in order to further evaluate and characterize the response of NF-κB to doxorubicin, specifically focusing on determining whether the induced activation of NF-κB is pro- or anti-apoptotic. To that end, in this study, we demonstrate that doxorubicin-induced repression of certain anti-apoptotic genes occurs through a mechanism that is independent of canonical NF-κB activity. Moreover, we show that doxorubicin-induced NF-κB activity in sarcomas is transcriptionally active and that targeted inhibition of canonical NF-κB activation enhances the cytotoxic effects of doxorubicin through increased apoptotic cell death.

## Results

### Compound A blocks doxorubicin-induced activation of canonical NF-κB

**Figure 1:**
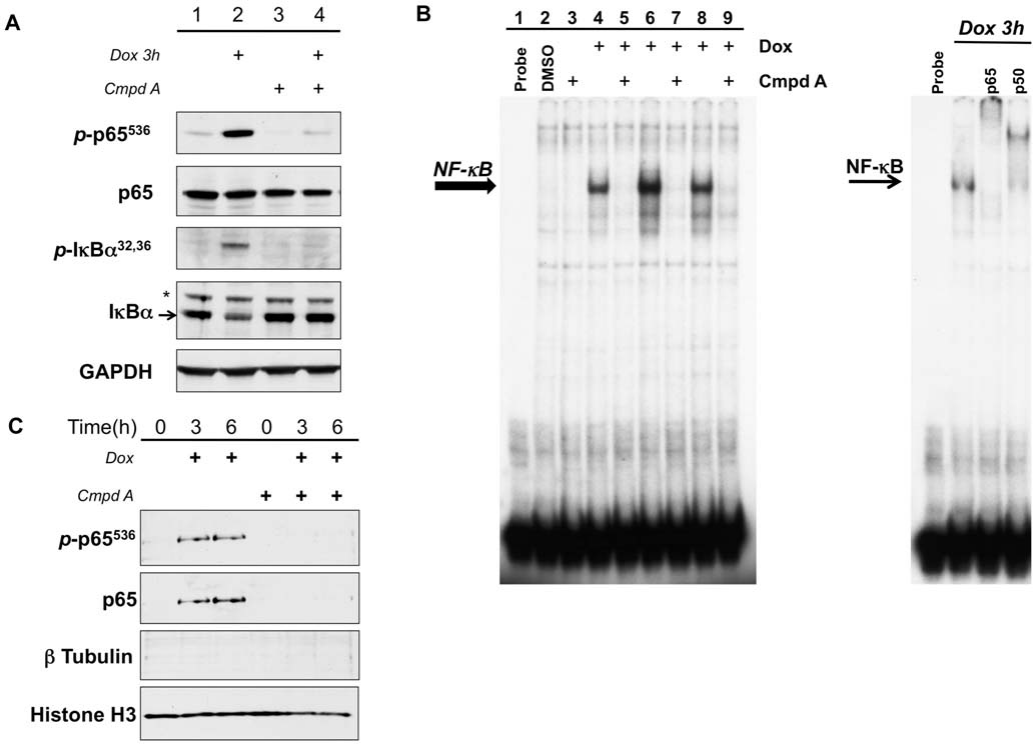
Doxorubicin activates canonical NF-κB signaling. (A) U2OS cells were pretreated with Compound A (Cmpd A, 5 µM) for 1 h and then stimulated with doxorubicin (Dox, 2 µM) for 3 h. Whole cell extracts were evaluated for canonical NF-κB activation by western blot. Doxorubicin induces phosphorylation of IκBα (*p*-IκBα^32,36^) and p65 (*p*-p65^536^) compared to DMSO-treated controls. Inhibition of the IKK complex with Cmpd A blocks doxorubicin-mediated increase in *p*-IκBα^32,36^ and *p*-p65^536^. (B) Nuclear extracts were prepared from U2OS cells treated with Dox+/−Cmpd A for 3, 6, and 12 hours and evaluated by EMSA. Doxorubicin treatment results in increased NF-κB DNA-binding activity at all times points. Pretreatment with Cmpd A successfully inhibits the activation of NF-κB by doxorubicin. Supershift analysis confirms that the activated complex of NF-κB contains both p65 and p50 subunits. (C) Western blot of nuclear extracts harvested from cells treated with Dox+/−Cmpd A demonstrates that doxorubicin induces nuclear translocation of p65 phosphorylated at serine 536 (*p*-p65^536^), and this is inhibited by the addition of Compound A.

Initially, we sought to characterize the effects of IKK inhibition using the small molecule IKKβ inhibitor [15], Compound A, on the ability of doxorubicin to activate NF-κB. To that end, U2OS cells were stimulated with doxorubicin (2 µM) for 3 hours with or without a one hour pretreatment with Compound A (5 µM). Cells treated with DMSO served as controls. Activation of the canonical NF-κB pathway was assessed by measuring the phosphorylation of IκBα at the serine 32 and 36 residues (*p*-IκBα^32,36^). Additionally, we evaluated the phosphorylation status of p65 at the serine 536 residue (*p*-p65^536^) as this modification has been shown to be important for transcriptional activity [1]. We found that treatment with doxorubicin resulted in a significant increase in *p*-IκBα^32,36^ (Figure 1A). This corresponded to a decrease in total levels of IκBα, which is consistent with proteasomal degradation (Figure 1A). Simultaneously, we determined that doxorubicin stimulation generated a substantial increase in *p*-p65^536^ when compared to untreated controls (Figure 1A). However, in cells pretreated with Compound A there was complete inhibition of doxorubicin-induced phosphorylation of both IκBα and p65 (Figure 1A). In order to confirm that doxorubicin can induce phosphorylation of p65 following 3 hours of treatment, we also examined this response in HT1080 fibrosarcoma cells. Similar to the response seen in U2OS cells, doxorubicin did induce a significant increase in *p*-p65^536^ at both 1.5 and 3-hour time points (Figure S1). Together these results demonstrate that doxorubicin activates the canonical NF-κB pathway and that targeting the IKK complex with Compound A efficiently blocks doxorubicin-induced NF-κB activity. Moreover, contrary to previously published work [11], doxorubicin treatment of U2OS cells induces phosphorylation at the serine 536 residue on p65 measured at 3 hours post treatment, suggesting that it may be transcriptionally active (see discussion).

### Compound A inhibits NF-κB DNA–binding activity and nuclear translocation of p65

Understanding that doxorubicin activates NF-κB downstream of the IKK complex, we then analyzed the effects of IKK inhibition with Compound A on the nuclear activity of NF-κB in response to doxorubicin. U2OS cells were treated with doxorubicin with or without Compound A for 3, 6 and 12 hours and nuclear extracts were isolated. We then assessed NF-κB DNA-binding activity *in vitro* using EMSA. We found that treatment with doxorubicin results in a robust increase in NF-κB DNA-binding activity at all time points compared to the DMSO-treated controls (Figure 1B, Lanes 4, 6, and 8). Supershift analysis confirmed that this activated complex contained both p65 and p50 subunits (Figure 1B). Importantly, when cells were pretreated with Compound A for one hour, doxorubicin-induced NF-κB DNA-binding activity was strongly inhibited (Figure 1B, Lanes 5, 7, and 9).

In order to confirm the results of the EMSA, we also examined the effects of doxorubicin treatment on the levels of nuclear p65. As above, U2OS cells were stimulated with doxorubicin in the presence or absence of Compound A and nuclear extracts were isolated. These extracts were then evaluated by western blot for the presence of p65. Consistent with the EMSA results, U2OS cells treated with doxorubicin for 3 and 6 hours had a significant increase in nuclear translocation of p65 when compared to DMSO-treated controls (Figure 1C). Again we noted the presence of increased phosphorylation at the serine 536 residue in cells exposed to doxorubicin (Figure 1C). When cells were pretreated with Compound A, the ability of doxorubicin to induce nuclear translocation of NF-κB was eliminated (Figure 1C). These results confirm that in response to doxorubicin, U2OS cells activate NF-κB leading to its accumulation in the nucleus. Moreover, these results further indicate that targeted inhibition of the IKK complex with Compound A is an effective means of blocking NF-κB nuclear activity, which is important for the analysis of downstream gene targets.

### NF-κB inhibition blocks doxorubicin-induced transcription of cIAP2, but does not alter doxorubicin-induced repression of other anti-apoptotic genes

**Figure 2:**
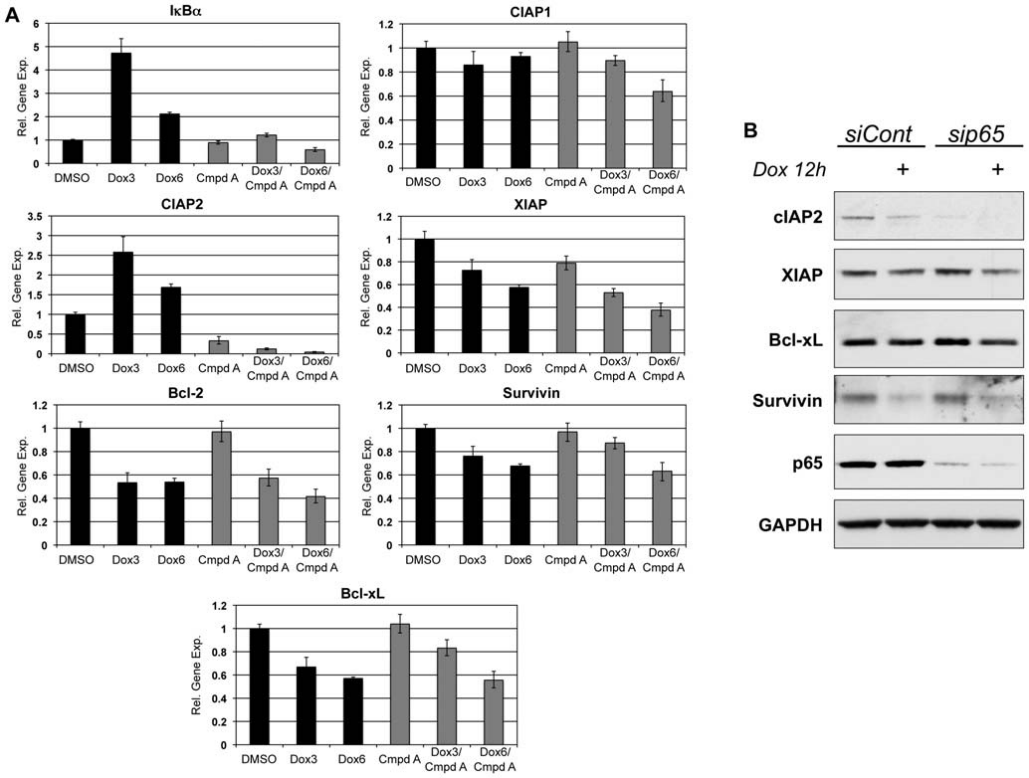
Doxorubicin-mediated repression of anti-apoptotic genes is independent of canonical NF-κB activity. (A) U2OS cells were stimulated with doxorubicin (Dox) with or without Compound A (Cmpd A) for the indicated times. Total RNA was isolated and evaluated using real time RT-PCR and results are displayed as relative gene expression compared to DMSO-treated controls. Doxorubicin treatment increased transcription of IκBα and cIAP2, decreased transcription of XIAP, Survivin, Bcl-xL, and Bcl2, and did not alter cIAP1 transcription (black bars). Inhibition of NF-κB activation with Cmpd A blocked the induction of IκBα and cIAP2, but had no effect on the doxorubicin-mediated repression of the other anti-apoptotic genes (gray bars). (B) U2OS cells were transfected with either a non-targeting control siRNA (siCont) or siRNA directed against p65 (sip65). After 48 h incubation, the cells were treated with doxorubicin (Dox) for 12 hours and changes in levels of anti-apoptotic proteins were assessed by western blot. Knockdown of the p65 subunit decreased the basal expression of cIAP2, but did not alter baseline protein levels of XIAP, Bcl-xL, or Survivin. Moreover, the absence of p65 did not alter the ability of doxorubicin to repress the expression of these same proteins.

We next evaluated the outcome of doxorubicin-induced NF-κB activity on gene expression. Following treatment with doxorubicin alone or in combination with Compound A for 3 and 6 hours, total RNA was extracted and evaluated for mRNA levels of potential NF-κB target genes by real time RT-PCR. First, we analyzed the effects of doxorubicin treatment on the transcription of IκBα, as it is directly regulated by NF-κB. Following three hours of doxorubicin stimulation, we determined that the mRNA levels of IκBα were increased nearly 5-fold compared to DMSO-treated controls (Figure 2A). By 6 h, the transcriptional activation began to subside and only a 2-fold elevation in IκBα mRNA was identified. This pattern of induction corresponds to the strong induction of p65 phosphorylation described above (Figure 1A and 1C). Furthermore, co-treatment with the IKK inhibitor completely abolished the doxorubicin-induced increase in IκBα transcription (Figure 2A). These results illustrate that the induction of IκBα transcription by doxorubicin is regulated by NF-κB, confirming that doxorubicin-induced NF-κB is transcriptionally active and is not globally repressive in its function.

To further evaluate the transcriptional activity of NF-κB in response to doxorubicin, we analyzed the expression patterns of a panel of anti-apoptotic genes in these cells. We selected genes that belong to both the Inhibitor of Apoptosis Protein (IAP) family and the Bcl-2-like protein family as they have been reported to be NF-κB regulated, both positively and negatively, in various cellular model systems [3]. Interestingly, we determined that doxorubicin treatment had varying effects on these genes. While there was no effect of doxorubicin on the expression of cIAP1 compared to DMSO-treated controls, there was a significant 2.5-fold induction of cIAP2 mRNA transcription following 3 hours of treatment (Figure 2A). On the other hand, when we examined other IAPs, we found that exposure to doxorubicin resulted in transcriptional repression of XIAP and Survivin (Figure 2A). Similarly, doxorubicin's ability to silence gene expression was also seen with Bcl-2 and Bcl-xL (Figure 2A), consistent with the results seen by Campbell et al [11].

However, in cells pretreated with Compound A prior to doxorubicin stimulation, we discovered that NF-κB inhibition had no effect on basal expression of cIAP1, although combination treatment did result in a modest decrease in cIAP1 mRNA (Figure 2A). When we evaluated cIAP2, we found that the addition of Compound A successfully inhibited the doxorubicin-induced increase in transcription, in addition to substantially decreasing basal mRNA levels (Figure 2A). Contrary to these results, combining NF-κB inhibition with doxorubicin did not alter the pattern of transcriptional repression of XIAP, Survivin, Bcl-2, or Bcl-xL seen in cells treated with doxorubicin alone (Figure 2A). These results provide further evidence that NF-κB is transcriptionally active in response to doxorubicin as evidenced by the regulation of cIAP2, which mirror the results seen with IκBα. Moreover, our data reveal that the ability of doxorubicin to cause transcriptional repression of the anti-apoptotic genes XIAP, Survivin, Bcl-2 and Bcl-xL is not dependent on NF-κB activity, at least that regulated by IKKβ.

In order to expand on these results and further evaluate if the effects at the mRNA level translated to protein expression, we selectively depleted the p65 subunit in U2OS cells using siRNA and then evaluated the effects of doxorubicin treatment on anti-apoptotic proteins. Cells transfected with a non-targeting siRNA (siCont) served as controls. The successful depletion of p65 was confirmed using western blot (Figure 2B). Interestingly, the increased transcription of cIAP2 did not translate to increased protein levels following exposure to doxorubicin. However, in the absence of p65, the levels of cIAP2 were significantly decreased basally and in response to doxorubicin (see Discussion). On the other hand, similar to the transcriptional results seen with Compound A, the knockdown of p65 did not alter the baseline protein expression of XIAP, Survivin, or Bcl-xL (Figure 2B). Moreover, treatment with doxorubicin resulted in decreased levels of these three anti-apoptotic genes and this pattern was unchanged in cells lacking the p65 subunit (Figure 2B). These results in combination with the transcriptional results seen using IKK inhibition support a role for NF-κB in both basal and doxorubicin-induced expression of cIAP2, and further suggest that NF-κB canonical activity is not mechanistically important for doxorubicin-induced repression of anti-apoptotic genes such as XIAP, Survivin, and Bcl-xL.

### NF-κB inhibition enhances the cytotoxicity of doxorubicin through increased apoptosis

**Figure 3:**
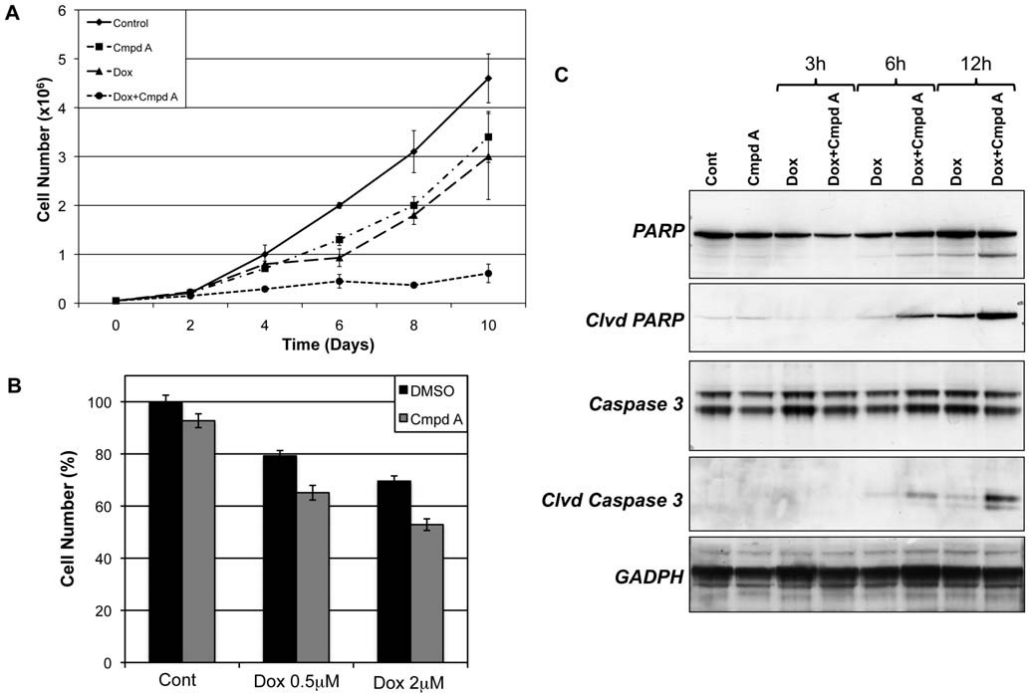
NF-κB inhibition with Compound A enhances the cytotoxicity of doxorubicin through increased apoptosis. (A) U2OS cells were seeded in 6-well plates and treated every 48 hours with DMSO, doxorubicin (Dox, 5 ng/ml), Compound A (Cmpd A, 5 µM), or Dox+Cmpd A. The total number of live cells was counted at 2-day intervals. While both Dox alone and Cmpd A alone resulted in some decrease in cell growth, the combination of NF-κB inhibition with doxorubicin substantially enhanced the cytotoxic effects of doxorubicin alone. (B) U2OS cells were pretreated with DMSO or Cmpd A and then exposed to Dox at either 0.5 µM or 2 µM for 24 hours. The number of cells was then measured using an MTS assay and displayed as the percent of total cells compared to DMSO-treated controls. Although Cmpd A alone had minimal effect on cell number, its combination with Dox at either dose resulted in decreased number of cells compared to doxorubicin alone. (C) U2OS cells treated with Dox+/−Cmpd A for the indicated time points were evaluated for the presence of the apoptotic markers cleaved PARP and cleaved Caspase 3. Treatment with Dox alone had minimal effects on apoptosis. However, the combination of NF-κB inhibition and Dox resulted in a significant increase in both cleaved PARP and cleaved Caspase 3, which is consistent with increased apoptotic cell death.

Having established that NF-κB activity has a selective transcriptional effect on cIAP2 and no effect on the doxorubicin-induced repression of other anti-apoptotic genes, we then explored the outcome of NF-κB inhibition on the cytotoxicity of doxorubicin. First, we evaluated the effects of combined treatment with doxorubicin and Compound A on cell growth and viability. A growth study was conducted using four treatment groups, DMSO, Compound A, doxorubicin, and doxorubicin plus Compound A. U2OS cells were seeded in 6-well plates and treated every 48 hours. The total number of live cells was then counted every 2 days using trypan blue staining. Although treatment with Compound A or doxorubicin individually had a modest effect on cell growth early, it appeared only to delay cell growth (Figure 3A). However, combination treatment with doxorubicin and Compound A resulted in a significant decrease in cell number that persisted throughout the course of the study (Figure 3A).

In order to further evaluate the outcome of combining NF-κB inhibition with doxorubicin, U2OS cells were seeded in a 96-well plate and subjected to treatment with DMSO, Compound A (5 µM), doxorubicin (0.5 µM or 2 µM), or doxorubicin plus Compound A for 24 hours. The overall number of cells was then quantified using an MTS assay. Treatment with Compound A alone had no effect on the overall number of cells compared to controls, while doxorubicin demonstrated a dose-dependent decrease in cell proliferation (Figure 3B). Consistent with the results of the growth study, when U2OS cells were pretreated with Compound A prior to the addition of doxorubicin there was a greater decrease in cell number compared to cells treated with doxorubicin alone (Figure 3B). Moreover, by incorporating NF-κB inhibition with the chemotherapy, equivalent cytotoxic effects of doxorubicin alone at 2 µM could be achieved using 75% less chemotherapeutic drug (Figure 3B).

Finally, we analyzed U2OS cells treated with either doxorubicin alone or in combination with Compound A for the presence of cleaved poly (ADP-ribose) polymerase (PARP) and cleaved Caspase 3 to determine if increased apoptosis was a component of the enhanced cytotoxicity. While doxorubicin was able to induce low levels of apoptosis by 12 hours, the combination of NF-κB inhibition and doxorubicin resulted in a substantial increase in the accumulation of cleaved PARP and cleaved Caspase 3 (Figure 3C). These results support a role for NF-κB activation in the inherent chemoresistance of osteosarcomas. Understanding that cIAP2 was regulated by NF-κB and induced by doxorubicin, we questioned whether this anti-apoptotic gene alone enabled U2OS cells to evade the cytotoxic effects of doxorubicin. However, selective depletion of cIAP2 using siRNA did not result in increased apoptosis in cells treated with doxorubicin (data not shown and see Discussion). The combination of these assays illustrates that increased NF-κB activity seen in response to doxorubicin inhibits the cell killing potential of doxorubicin.

### IKKα and IKKβ are critical to doxorubicin-induced NF-κB activity

**Figure 4:**
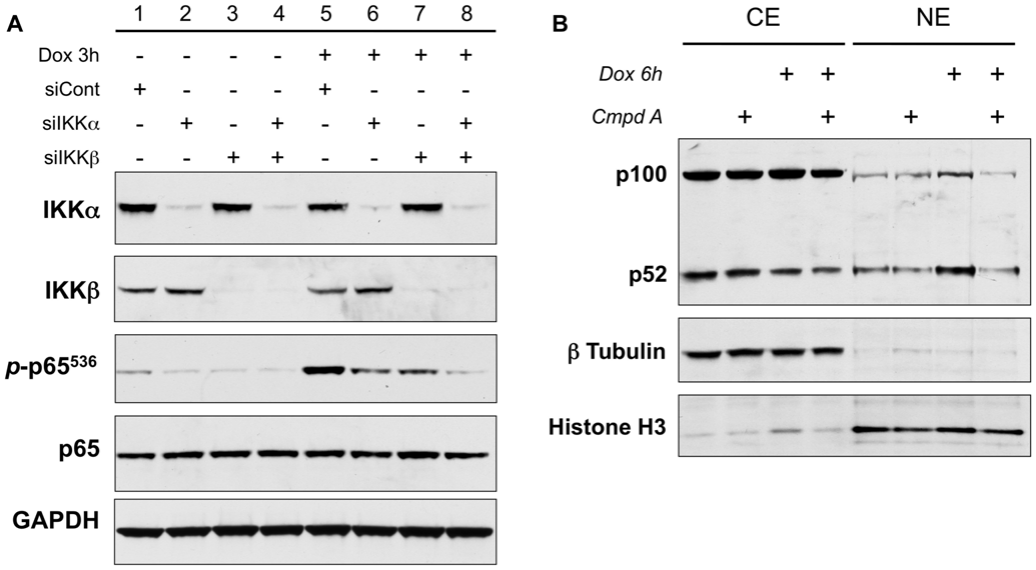
Both IKKα and IKKβ contribute to doxorubicin-induced phosphorylation of p65 and doxorubicin induces nuclear accumulation of p52. (A) U2OS cells were transfected with siRNA constructs targeting IKKα (siIKKα), IKKβ (siIKKβ) or a non-targeting control siRNA (siCont). The cells were then treated with doxorubicin (Dox) for 3 hours. Western blot confirmed that siIKKα and siIKKβ achieved selective knockdown of their respective subunits. Analysis of phosphorylation of p65 (*p*-p65^536^) demonstrated that the decrease in either IKKα or IKKβ alone did not completely eliminate the ability of doxorubicin to induce *p*-p65^536^. However, knockdown of both catalytic subunits resulted in complete inhibition of the doxorubicin-induced increase in p65 phosphorylation. (B) Cytoplasmic and nuclear extracts were isolated from U2OS cells treated with doxorubicin (Dox) +/− Compound A (Cmpd A) for 6 hours. Immunoblot for p52/p100 confirms an increase in the nuclear translocation of p52 in response to Dox treatment. Inhibition of the IKK complex with Compound A prevented the nuclear accumulation of p52.

In order to further explore the mechanism behind NF-κB-mediated chemoresistance, we initially further dissected the means by which doxorubicin activates NF-κB. Based on previous results from our laboratory showing an important role for both IKKα and IKKβ in the NF-κB response to doxorubicin [16], we hypothesized that similar functions of these kinases existed in osteosarcomas. To address this question, U2OS cells were transfected with siRNA targeting the IKKα subunit, the IKKβ subunit, or both. A non-targeting siRNA (siCont) served as a control. The cells were incubated for 48 hours and then stimulated with doxorubicin for 3 hours. Whole cell extracts were then evaluated for phosphorylation of p65 at the serine 536 residue. We discovered that the knockdown of either IKKα or IKKβ resulted in diminished levels of doxorubicin-induced *p*-p65^536^ when compared to cells transfected with siCont (Figure 4A). However, in the absence of either catalytic subunit alone, doxorubicin was able to generate a modest increase in NF-κB activation compared to untreated controls, but targeting both subunits completely prevented the induction of *p*-p65^536^ (Figure 4A). Consistent with our previous work in fibrosarcomas, doxorubicin activates canonical NF-κB signaling through both IKKα and IKKβ. Although it has been recently shown that IKKα can activate the canonical pathway as a compensatory mechanism in the absence of IKKβ [17], our data demonstrates that in sarcomas both catalytic subunits mutually contribute to the activation of NF-κB in response to doxorubicin.

### Doxorubicin induces nuclear accumulation of p52

Understanding that IKKα actively participates in the canonical NF-κB response to doxorubicin, we proceeded to evaluate the effects of doxorubicin on the alternative NF-κB pathway. Specifically, we treated U2OS cells with doxorubicin (2 µM) in the presence or absence of Compound A (5 µM) for 6 hours. Cytoplasmic and nuclear extracts were then prepared and evaluated for the presence of p52, a critical component and effector of the alternative NF-κB pathway. Interestingly, we found that treatment with doxorubicin did increase the levels of p52 in the nucleus when compared to DMSO-treated controls (Figure 4B). This nuclear accumulation of p52 was effectively inhibited when cells were pretreated with Compound A (Figure 4B, see Discussion).

### Activation of p65, not p52, is critical to chemoresistance of sarcomas

**Figure 5:**
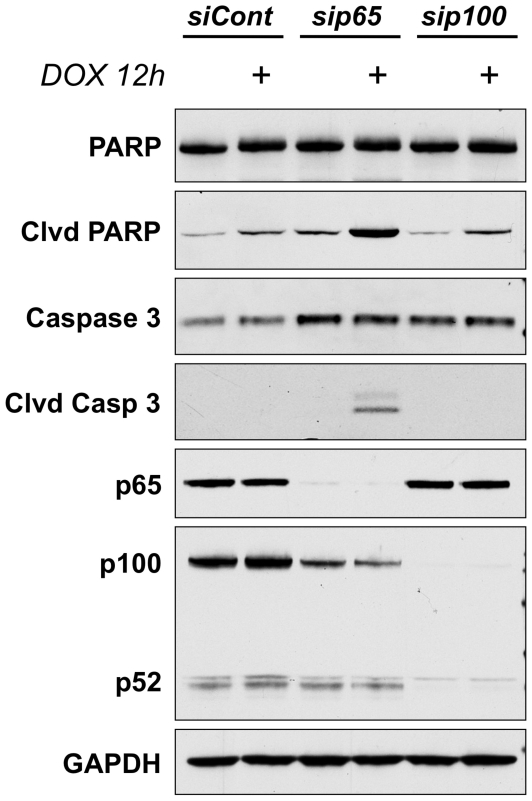
Activation of p65, and not p52, is critical to the apoptotic resistance in U2OS cells. U2OS cells were transfected with siRNA targeting the p65 subunit (sip65), the p100 subunit (sip100), or a non-targeting control (siCont). After 48 hours of incubation with the siRNA, the cells were treated with doxorubicin for 12 hours and the effects on apoptosis were assessed by western blot. Western blot for p65 confirmed significant knockdown. Evaluation of p100 levels revealed a modest decrease of p100 in the cells lacking p65 and complete knockdown in the cells treated with sip100. Importantly, the levels of p52 were not affected by sip65, but were substantially decreased in the absence of its precursor p100. Analysis of the apoptotic markers cleaved PARP and cleaved Caspase 3, revealed a robust increase in apoptosis in response to doxorubicin only in cells lacking the p65 subunit. Cells treated with siCont or sip100 demonstrated no increase in cleaved PARP or cleaved Caspase 3 when treated with doxorubicin.

Next we evaluated the individual contributions of each NF-κB subunit to the chemoresistance of osteosarcomas. To do so we transfected U2OS cells with siRNA targeting the p65 subunit, the p100 subunit (the precursor for p52), or a non-targeting control. The cells were then treated with doxorubicin for 12 hours and assessed for the onset of apoptosis. Western blots confirmed that adequate and selective knockdown of the individual subunits was achieved (Figure 5). Treatment with doxorubicin alone was unable to induce measurable apoptosis (Figure 5). The results were similar when cells lacking p100/p52 were stimulated with doxorubicin (Figure 5). However, in cells in which p65 was depleted, exposure to doxorubicin resulted in a substantial increase in levels of cleaved PARP and cleaved Caspase 3 indicating ongoing apoptosis (Figure 5). The inability of sip100 to enhance doxorubicin-induced cell death, demonstrates that the enhanced nuclear accumulation of p52 does not directly contribute to the U2OS cells' resistance to the apoptotic effects of chemotherapy. On the other hand, the p65 subunit has an active role in the ability of osteosarcomas to resist the cytotoxic effects of doxorubicin, highlighting the importance of canonical NF-κB signaling as a critical mechanism underlying the chemoresistance of sarcomas.

## Discussion

Our study demonstrates an important role for NF-κB activity in the ability of osteosarcomas to resist the cytotoxicity of the commonly used chemotherapeutic, doxorubicin. In contrast to previously published work [11], we show that in response to doxorubicin, a transcriptionally active form of p65 is generated and accumulates in the nucleus. This in turn leads to the increased transcription of cIAP2 and IκBα. We also illustrate that the repression of several anti-apoptotic genes by doxorubicin occurs in a manner independent of canonical NF-κB activity. Importantly, the combination of canonical NF-κB inhibition with doxorubicin resulted in enhanced cytotoxicity compared to doxorubicin alone, in part through increased apoptotic cell death.

Previously, others have reported that doxorubicin or its analogue daunorubicin activates NF-κB in such a manner that it recruits p65 to repress its target genes. In those studies, there is increased DNA-binding activity by EMSA in response to drug treatment, but no change is identified in the level of phosphorylated p65 at the serine 536 residue [11], [12], which has been established as a important modification for NF-κB transcriptional activity [1]. In contrast to the results seen by Campbell et al. after 5 hours of treatment with chemotherapy [11], in our study we identified a significant increase in p65 phosphorylation at serine 536 following 3 hours of treatment with doxorubicin in both U2OS and HT1080 cells. This difference in exposure to doxorubicin suggests that an earlier assessment of response may be critical to identify changes in p65 phosphorylation. The importance of early evaluation of treatments on NF-κB activity is also evident in the assessment of transcriptional targets, as this early increase in *p*-p65^536^ did correlate with increased transcription of IκBα and cIAP2. On the other hand, Ho et al. demonstrated that doxorubicin treatment in MDA-MB-231 breast cancer cells did not increase p65 phosphorylation at serine 536 at any time from 10 minutes up to 24 hours and concordantly demonstrated a decrease in cIAP2 transcription [12]. Similarly, we also did not identify any alterations in *p*-p65^536^ in these breast cancer cells after 1.5 or 3 hours of doxorubicin treatment (Figure S1). Therefore, identifying the presence or absence of this modification could serve as a marker to determine whether canonical NF-κB activated by chemotherapeutics is transcriptionally active or repressive, respectively.

In addition to the absence of post-translational phosphorylation at serine 536, reports in the literature supporting a repressive function for NF-κB have relied on evaluating a limited subset of anti-apoptotic genes and correlating those expression patterns with luciferase reporters [11], [12], [18]. However, this correlation can be difficult in the setting of transient activation of NF-κB and time required to accumulate firefly luciferase for an accurate reading. For instance in U2OS cells, we reveal that the induction of transcription of IκBα and cIAP2 is identified early following 3 hours of doxorubicin treatment, and subsequently declines. Under such conditions, the use of a luciferase reporter assay, which requires sufficient treatment times to allow for translation and accumulation of firefly luciferase, may not accurately depict the subtle changes at individual promoters at shorter time points. This issue is evident in our study, where despite identifying increased mRNA levels for two NF-κB target genes, the NF-κB luciferase reporter assay shows decreased expression of the reporter gene in cells treated with doxorubicin (Figure S2). Additionally, if the mechanism by which doxorubicin represses gene transcription is not regulated by NF-κB, it may also silence the transcription of exogenous luciferase reporters.

Moreover, reports that link NF-κB activity to the repression of anti-apoptotic genes lack substantial evidence examining the overall effects of NF-κB inhibition on the cytotoxicity of doxorubicin, which is an important question in regards to future clinical applications [11], [12]. As a result, in the present study we incorporated several techniques to assess the outcome of combining targeted NF-κB inhibition with chemotherapy on cell growth, cell number and apoptosis. Using the IKKβ inhibitor, Compound A, we illustrate that blocking the induction of NF-κB activity can enhance the efficacy of doxorubicin, as evidenced by decreased cell growth and increased levels of cleaved PARP and cleaved Caspase 3. We then further confirmed that canonical NF-κB signaling is important to the chemoresistance of U2OS cells by showing similar increases in apoptosis when cells were first depleted of p65 using siRNA prior to treatment with doxorubicin. Based on these results, NF-κB is anti-apoptotic in its function in response to doxorubicin and inhibiting the activation of canonical NF-κB is an effective means for overcoming the chemoresistance in sarcomas.

However, the mechanism underlying the anti-apoptotic function of NF-κB in U2OS cells remains elusive. In light of the fact that selective depletion of cIAP2 did not generate enhanced apoptosis (data not shown), the classical model of NF-κB-mediated induction of anti-apoptotic genes may not be sufficient, in and of itself, to explain the added benefits seen in the combination treatment groups. Further studies are ongoing to determine the key targets downstream of canonical NF-κB activation that are responsible for mediating the resistance to doxorubicin-induced cell death in sarcomas.

Given the active role of IKKα in the canonical NF-κB response to doxorubicin, it will be important to further identify the contributions of p52 activation to the ability of sarcoma cells to evade the toxicity of standard chemotherapy. In our study we demonstrate that doxorubicin treatment induces an increase in nuclear accumulation of p52, and we further show that IKK inhibition with Compound A abrogates this response. Further studies will be necessary to determine if the increase in nuclear p52 is the result of true activation of the non-canonical pathway through IKKα-mediated phosphorylation of p100 and its subsequent cleavage to p52, or if there are other mechanisms downstream of IKKα and IKKβ that are driving additional NF-κB subunits to the nucleus. In addition to determining the mechanism leading to increased nuclear p52, it will be important to identify its contribution to chemoresistance in response to doxorubicin. Although we demonstrate that its knockdown in isolation did not enhance cell death, p52 may contribute in other ways to the ability of U2OS cells to evade cytotoxic effects of chemotherapy. Specifically, further investigation of the potential role of p52 in the regulation of the cell cycle in osteosarcoma cells treated with doxorubicin will be important as others demonstrated p52's ability to influence the expression of several key cell cycle regulators such as Cyclin D1 and p21 [19]–[21].

Furthermore, as the drive to design and discover new cancer therapeutics continues, it is important to realize the importance of the IKKα subunit in cancer chemoresistance. To date, the majority of research on targeted inhibition has focused on the IKKβ subunit because of its role in NF-κB activation by cytokines [2]. In this study, we demonstrate that selective depletion of either IKKα or IKKβ alone is not sufficient to inhibit the activation of canonical NF-κB activity. Additionally, treatment with the IKKβ inhibitor Compound A at lower doses (1 µM) was unable to completely inhibit the activation of canonical NF-κB (data not shown). However, by increasing the concentration of Compound A (5 µM), we were able to completely block doxorubicin-induced canonical NF-κB activity. Given that Ziegelbauer et al. demonstrated that although Compound A is more selective for IKKβ it also effectively inhibits IKKα at higher concentrations [15], we hypothesize that Compound A at higher doses targets both IKKα and IKKβ and therefore can prevent downstream activation of canonical NF-κB signaling by doxorubicin. The combination of these results, together with our previous work [16], highlights an important and active role for IKKα in chemotherapy-induced activation of canonical NF-κB activity. Moreover, Lam et al have recently shown that IKKα can be recruited to activate p65 as a compensatory response in the absence of IKKβ [17]. Therefore, achieving adequate and complete inhibition of NF-κB activation as an adjunct to conventional chemotherapy is likely to require successful targeting of both subunits, rather than isolated inhibition of IKKβ alone.

For malignancies resistant to standard chemotherapies like sarcomas, where the chemotherapeutic options have not changed in nearly 20 years, nor has the 12–16 month median survival for patients with metastatic disease, the concept of combating the inherent chemoresistance of solid tumors with novel biologic therapies has never been more important [13]. Our results demonstrate that the transcription factor NF-κB actively influences the cellular response to chemotherapy. Therefore, combination therapies incorporating NF-κB inhibitors, such as small molecules targeting the IKK complex, together with standard chemotherapies remains a viable method to improve the clinical outcomes in patients with advanced stage malignancies.

## Materials and Methods

### Cell culture and reagents

U2OS human osteosarcoma cells, HT1080 human fibrosarcoma cells, and MDA-MB-231 breast cancer cells were obtained form American Type Culture Collection (ATCC, Rockville, MD). The U2OS cells, HT1080 cells and MDA-MB-231 cells were maintained in McCoy's 5A medium (Mediatech, Inc., Manassas, VA), MEM-Alpha medium, and DMEM medium (Invitrogen, Carlsbad, CA), respectively. All growth media was supplemented with 10% fetal bovine serum and 100 µg/ml penicillin and 100 µg/ml streptomycin. Cell cultures were maintained at 37°C with a mixture of 95% air and 5% CO2. Cells were treated with doxorubicin (1 µg/ml) and harvested at the indicated time points. Inhibition of NF-κB was accomplished using a small molecule inhibitor of IKKβ (Compound A) obtained from Theralogics (Chapel Hill, NC). It can target both IKKα and IKKβ subunits, but exhibits a greater affinity for IKKβ [15]. Cells were pretreated with Compound A for 1 hour prior to stimulation with doxorubicin. For all experiments incorporating treatment with Compound A, cells treated with DMSO served as a control.

### Small interference RNA (siRNA) transfection

In preparation for transfection 2.5×10^5^ cells were plated in 6-well plates and incubated overnight. The transfections were then carried out according to the manufacturer's protocol. Briefly, the growth media was removed and replaced with transfection media containing siGENOME SMARTpool siRNA for siControl #4 or #5, sip65, sip100, siIKKα, or siIKKβ (final concentration 20 nM) and DharmaFECT1 transfection reagent (Dharmacon, Inc., Lafayette, CO). For knockdown of both IKKα and IKKβ, 20 nM of siRNA targeting each individual subunit was added to the transfection medium. The cells were cultured for 24 hours. The transfection media was then removed and replaced with standard growth media. The cells were grown for an additional 24 hours and treated with doxorubicin as described above. The knockdown of target proteins was confirmed with western blot.

### Western blot and antibodies

Cytoplasmic and nuclear extracts were prepared as previously described [16]. Briefly, cytoplasmic extracts were isolated using a hypotonic buffer [10 mM Hepes (pH 7.6), 60 mM KCL, 1 mM EDTA, 1 mM DTT, 0.2% NP-40, 1 mM PMSF] and centrifugation. The nuclei were then suspended in a high-salt buffer (20 mM Tris pH 8.0, 420 mM NaCl, 1.5 mM MgCl2, 0.2 mM EDTA, 1 mM PMSF, 25% glycerol) for ten minutes and again separated from cellular debris using centrifugation. For analysis of whole cell extracts, cells were suspended in a lysis buffer [20 mM Tris-HCL (pH 7.5), 150 mM NaCl, 1 mM Na2EDTA, 1 mM EGTA, 1% Triton, 2.5 mM sodium pyrophosphate, 1 mM β-glycerophosphate, 1 mM Na_3_VO_4_] for 5 minutes. The lysate was then separated using centrifugation at 13,000rpm for 10 minutes. All lysis buffers were supplemented with protease inhibitors (Roche Diagnostics, Mannheim, Germany) and phosphatase inhibitors (Sigma Aldrich, St. Louis, MO) Protein concentrations of the lysates were determined using a Bradford Protein assay.

Western blot analysis was performed following the protocol provided by Invitrogen (Carlsbad, CA) by separating proteins (20–30 µg) on NuPAGE 4–12% Bis-Tris gels. Antibodies for IκBα, β-Tubulin, p65, survivin (Santa Cruz Biotechnology, Inc., Santa Cruz, CA), caspase 3, cleaved caspase 3, cleaved PARP, PARP, phosphorylated p65^536^, phosphorylated IκBα^32,36^, p100/p52, histone H3, Bcl-xL, cIAP2, and XIAP (Cell Signaling Technology, Inc., Danver, MA), IKKα and IKKβ (Upstate) were used at 1∶1000 dilution. All antibodies were diluted in 5% bovine albumin (Sigma-Aldrich, St. Louis, MO) in TBS-T, except for phosphorylated IκBα^32,36^, which was diluted in 5% non-fat milk in TBS-T. The blots were incubated with the primary antibody overnight at 4°C. The membranes were then incubated with secondary anti-rabbit or anti-mouse antibody diluted at a range of 1∶5000 or 1∶10000 in 5% milk in TBS-T for 1 hour and then developed using Amersham ECL Western Blotting Detection Reagents (GE Healthcare, Buckinghamshire, England).

### Electrophoretic Mobility Shift Assay (EMSA)

Cells were harvested after treatment as indicated above, and the EMSAs performed as previously reported [22]. In brief, nuclear extracts were obtained as described above, and 5 µg of nuclear proteins were incubated with 1 µg/µl polydIdC in binding buffer (50 mM Tris pH 7.6, 5 mM DTT, 2.5 mM EDTA, 50% glycerol) for 15 minutes. Subsequently, an oligonucleotide radiolabeled with [α^32^P]dCTP was added and allowed to incubate for an additional 15 minutes at room temperature. The probe contains an NF-κB consensus binding site for the H-2κB promoter (5′-GGGGATTCCC-3′). The samples were then separated on a polyacrylamide gel and developed with autoradiography. For supershifts, 1 µl of p65X and p50X (200 µg/0.1 ml; Santa Cruz Biotechnology, Inc., Santa Cruz, CA) was added together with polydIdC for the initial incubation.

### Real time RT–PCR

After treatment, total RNA was isolated using Trizol (Invitrogen, Carlsbad, CA). Reverse transcription was conducted using 1 µg of total RNA using MMLV reverse transcriptase (Invitrogen, Carlsbad, CA) according to the manufacturer's protocol. Real time RT-PCR was then performed using TaqMan gene expression assays for IκBα, cIAP1, cIAP2, XIAP, Survivin, Bcl-2, and Bcl-xL (Applied Biosystems Inc., Forest City, CA) on an ABI 7900HT real time PCR system (Applied Biosystems, Inc., Forest City, CA).

### Growth study

2.5×10^4^ cells were seeded in 6-well plates and incubated for 24 hours. The media was then exchanged and the cells were treated with doxorubicin (5 ng/ml) +/− Compound A (5 µM) as described above. Again cells treated with DMSO served as controls. Media was replaced every 48 hours and the cells were again treated with doxorubicin +/− Compound A. This continued for a total of 10 days. Cells were trypsinized and counted using trypan blue at 2, 4, 6, 8, and 10 days. Experiments were conducted in triplicate and displayed as total number of live cells.

### MTS assay

U2OS cells (5×10^3^/well) were seeded in a 96-well plate and allowed to incubate for 24 hours. The cells were then pretreated with Compound A for 1 hour and then stimulated with doxorubicin for 24 hours, and DMSO treated cells served as controls. The MTS assay (Promega, Madison, WI) was then conducted according to the manufacturer's protocol. Briefly, 20 µl of the reagent were added to each well and the plates were incubated at 37°C for approximately 1.5 hours. The absorbance of each well at 490 nm was then measured on the VERSAmax microplate reader (Molecular Devices, Sunnydale, CA). Treatments were performed in triplicate and the results are displayed as the average percentage of total cell number.

### Dual luciferase assay

U2OS cells were seeded in 6-well plates at 2×10^5^ cells per well and cultured for 24 hours. Cells were then transfected with 250 ng/well of a 3×κB luciferase reporter construct together with 2.5 ng/well of pRL-TK Renilla luciferase construct (Promega, Madison, WI) using 2 µg polyethylenimine/µg of DNA. The cells were incubated overnight, media was exchanged and the cells were treated with DMSO, doxorubicin, Compound A, or doxorubicin + Compound A for 12 hours. The cells were then harvested in passive lysis buffer and analyzed using the Dual Luciferase Assay System according to the manufacturer's protocol (Promega, Madison, WI) on an Lmax Microplate Luminometer (Molecular Devices, Sunnydale CA). Relative light units of the 3×κB luciferase were normalized to Renilla luciferase light units to control for transfection efficiency. Experiments were performed in triplicate.

## Supporting Information

Figure S1 Doxorubicin induces phosphorylation of p65 in HT1080 cells. HT1080 fibrosarcoma cells and MDA-MB-231 breast cancer cells were treated with doxorubicin (DOX) for 1.5 and 3 hours. Whole cell lysates were then evaluated for the presence of phosphorylation of p65 at serine 536 (p65^536^). Doxorubicin treatment resulted in increased levels of p65^536^ at both time points in HT1080 cells, but did not alter the level of p65 phosphorylation in MDA-MB-231 cells.
(0.99 MB TIF)

Figure S2 Doxorubicin treatment represses NF-κB luciferase reporter. U2OS cells were transfected with both a 3×κB firefly luciferase reporter construct and a Renilla construct to serve as a control for transfection efficiency. After incubation for 24 hours, the cells were subsequently stimulated with doxorubicin (Dox) with or without Compound A (Cmpd A) for 12 hours. The cells were then lysed and evaluated using a dual luciferase assay. Treatment with doxorubicin resulted in repression of the NF-κB reporter when compared to DMSO treated controls. Additionally, Compound A alone or in combination with doxorubicin was also capable of silencing the NF-κB reporter.
(0.14 MB TIF)

## References

[1] Basseres DS, Baldwin AS (2006). Nuclear factor-kappaB and inhibitor of kappaB kinase pathways in oncogenic initiation and progression.. Oncogene.

[2] Kim HJ, Hawke N, Baldwin AS (2006). NF-kappaB and IKK as therapeutic targets in cancer.. Cell Death Differ.

[3] Shen HM, Tergaonkar V (2009). NFkappaB signaling in carcinogenesis and as a potential molecular target for cancer therapy.. Apoptosis.

[4] Wang CY, Mayo MW, Baldwin AS (1996). TNF- and cancer therapy-induced apoptosis: potentiation by inhibition of NF-kappaB.. Science.

[5] Wang CY, Guttridge DC, Mayo MW, Baldwin AS (1999). NF-kappaB induces expression of the Bcl-2 homologue A1/Bfl-1 to preferentially suppress chemotherapy-induced apoptosis.. Mol Cell Biol.

[6] Cusack JC, Liu R, Baldwin AS (2000). Inducible chemoresistance to 7-ethyl-10-[4-(1-piperidino)-1-piperidino]-carbonyloxycamptothe cin (CPT-11) in colorectal cancer cells and a xenograft model is overcome by inhibition of nuclear factor-kappaB activation.. Cancer Res.

[7] Cusack JC, Liu R, Houston M, Abendroth K, Elliott PJ (2001). Enhanced chemosensitivity to CPT-11 with proteasome inhibitor PS-341: implications for systemic nuclear factor-kappaB inhibition.. Cancer Res.

[8] Chen W, Wang X, Bai L, Liang X, Zhuang J (2008). Blockage of NF-kappaB by IKKbeta- or RelA-siRNA rather than the NF-kappaB super-suppressor IkappaBalpha mutant potentiates adriamycin-induced cytotoxicity in lung cancer cells.. J Cell Biochem.

[9] Gangadharan C, Thoh M, Manna SK (2009). Inhibition of constitutive activity of nuclear transcription factor kappaB sensitizes doxorubicin-resistant cells to apoptosis.. J Cell Biochem.

[10] Ashikawa K, Shishodia S, Fokt I, Priebe W, Aggarwal BB (2004). Evidence that activation of nuclear factor-kappaB is essential for the cytotoxic effects of doxorubicin and its analogues.. Biochem Pharmacol.

[11] Campbell KJ, Rocha S, Perkins ND (2004). Active repression of antiapoptotic gene expression by RelA(p65) NF-kappa B.. Mol Cell.

[12] Ho WC, Dickson KM, Barker PA (2005). Nuclear factor-kappaB induced by doxorubicin is deficient in phosphorylation and acetylation and represses nuclear factor-kappaB-dependent transcription in cancer cells.. Cancer Res.

[13] Thornton K (2008). Chemotherapeutic management of soft tissue sarcoma.. Surg Clin North Am.

[14] Hueman MT, Herman JM, Ahuja N (2008). Management of retroperitoneal sarcomas.. Surg Clin North Am.

[15] Ziegelbauer K, Gantner F, Lukacs NW, Berlin A, Fuchikami K (2005). A selective novel low-molecular-weight inhibitor of IkappaB kinase-beta (IKK-beta) prevents pulmonary inflammation and shows broad anti-inflammatory activity.. Br J Pharmacol.

[16] Bednarski BK, Ding X, Coombe K, Baldwin AS, Kim HJ (2008). Active roles for inhibitory kappaB kinases alpha and beta in nuclear factor-kappaB-mediated chemoresistance to doxorubicin.. Mol Cancer Ther.

[17] Lam LT, Davis RE, Ngo VN, Lenz G, Wright G (2008). Compensatory IKKalpha activation of classical NF-kappaB signaling during IKKbeta inhibition identified by an RNA interference sensitization screen.. Proc Natl Acad Sci U S A.

[18] Campbell KJ, O'Shea JM, Perkins ND (2006). Differential regulation of NF-kappaB activation and function by topoisomerase II inhibitors.. BMC Cancer.

[19] Nadiminty N, Chun JY, Lou W, Lin X, Gao AC (2008). NF-kappaB2/p52 enhances androgen-independent growth of human LNCaP cells via protection from apoptotic cell death and cell cycle arrest induced by androgen-deprivation.. Prostate.

[20] Rocha S, Martin AM, Meek DW, Perkins ND (2003). p53 represses cyclin D1 transcription through down regulation of Bcl-3 and inducing increased association of the p52 NF-kappaB subunit with histone deacetylase 1.. Mol Cell Biol.

[21] Schumm K, Rocha S, Caamano J, Perkins ND (2006). Regulation of p53 tumour suppressor target gene expression by the p52 NF-kappaB subunit.. Embo J.

[22] Mayo MW, Wang CY, Cogswell PC, Rogers-Graham KS, Lowe SW (1997). Requirement of NF-kappaB activation to suppress p53-independent apoptosis induced by oncogenic Ras.. Science.

