# Supplementary figures and images for: Addressing Reported Pro-Apoptotic Functions of NF-κB: Targeted Inhibition of Canonical NF-κB Enhances the Apoptotic Effects of Doxorubicin
Source: PLoS One. 2009 Sep 10;4(9):e6992. doi: 10.1371/journal.pone.0006992 (PMC2734988; doi:10.1371/journal.pone.0006992)

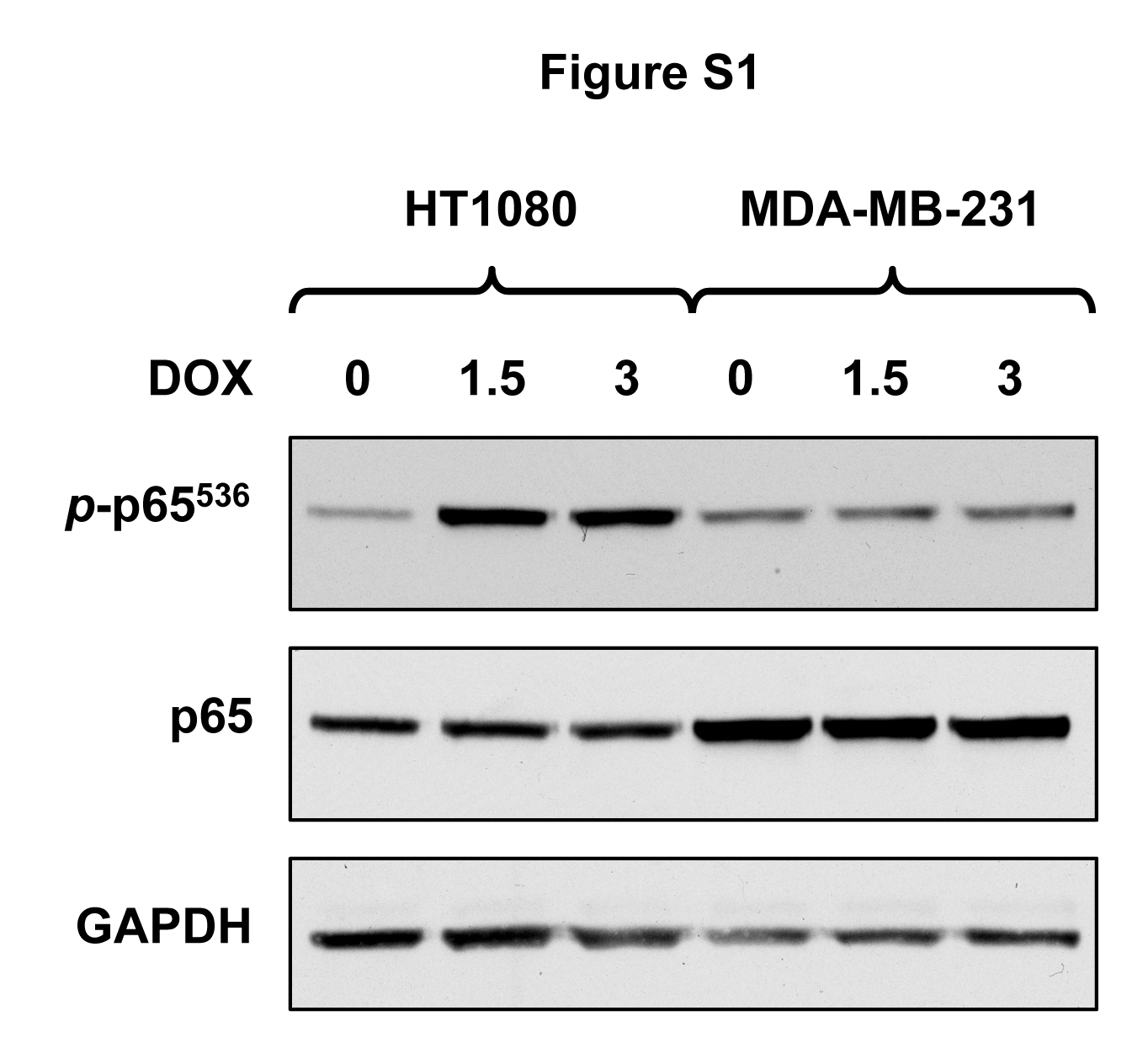

Supplement: Figure S1 — Doxorubicin induces phosphorylation of p65 in HT1080 cells. HT1080 fibrosarcoma cells and MDA-MB-231 breast cancer cells were treated with doxorubicin (DOX) for 1.5 and 3 hours. Whole cell lysates were then evaluated for the presence of phosphorylation of p65 at serine 536 (p65536). Doxorubicin treatment resulted in increased levels of p65536 at both time points in HT1080 cells, but did not alter the level of p65 phosphorylation in MDA-MB-231 cells. (0.99 MB TIF) [file pone.0006992.s001.tif]

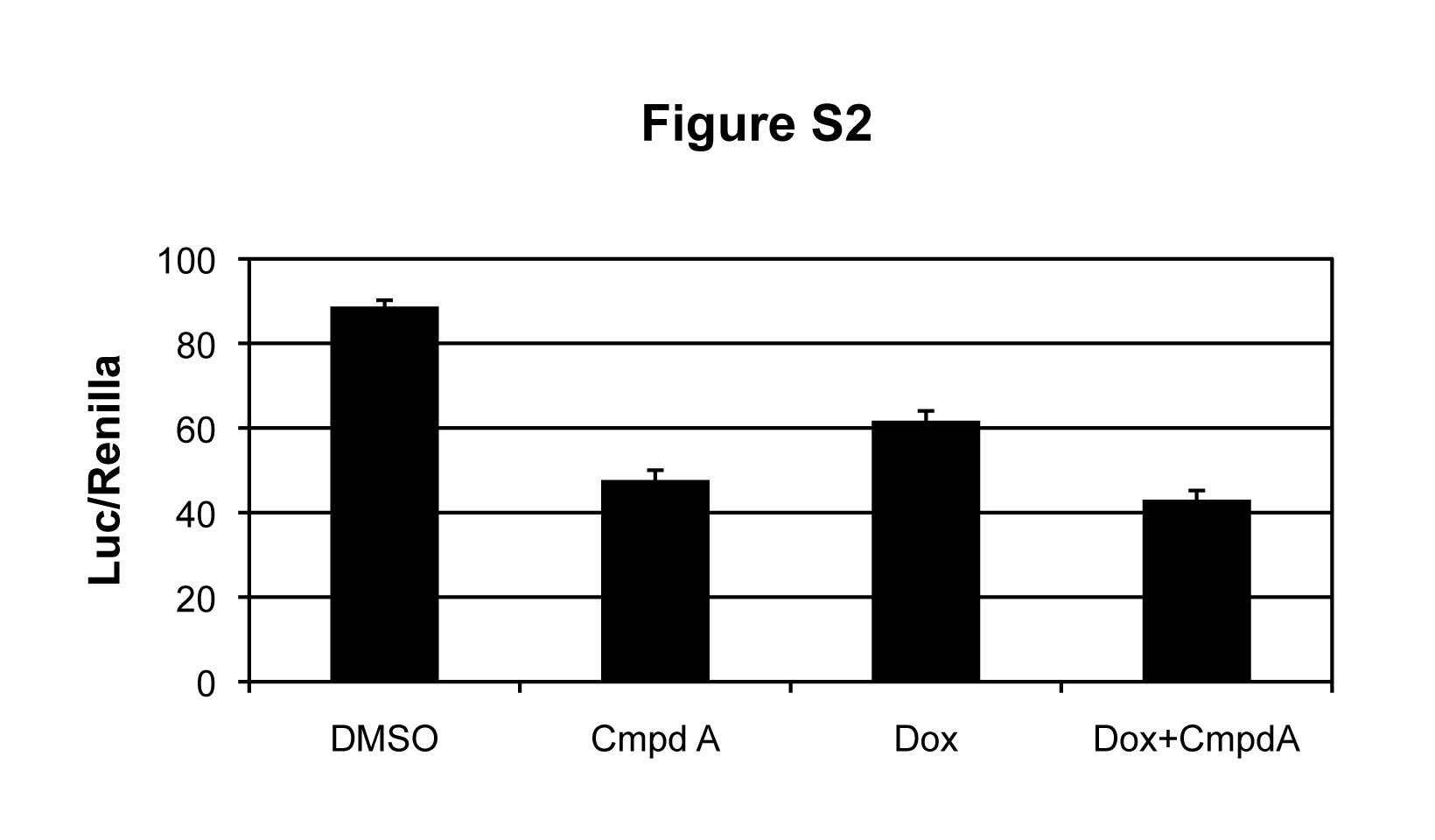

Supplement: Figure S2 — Doxorubicin treatment represses NF-κB luciferase reporter. U2OS cells were transfected with both a 3×κB firefly luciferase reporter construct and a Renilla construct to serve as a control for transfection efficiency. After incubation for 24 hours, the cells were subsequently stimulated with doxorubicin (Dox) with or without Compound A (Cmpd A) for 12 hours. The cells were then lysed and evaluated using a dual luciferase assay. Treatment with doxorubicin resulted in repression of the NF-κB reporter when compared to DMSO treated controls. Additionally, Compound A alone or in combination with doxorubicin was also capable of silencing the NF-κB reporter. (0.14 MB TIF) [file pone.0006992.s002.tif]
